# Antimicrobial Activity of Some Thiourea Derivatives and Their Nickel and Copper Complexes

**DOI:** 10.3390/molecules14010519

**Published:** 2009-01-22

**Authors:** Hakan Arslan, Nizami Duran, Gulay Borekci, Cemal Koray Ozer, Cevdet Akbay

**Affiliations:** 1 Department of Natural Sciences, Fayetteville State University, Fayetteville, NC 28301, USA; E-mail: cakbay@uncfsu.edu (C. A.); 2 Department of Chemistry, Faculty of Pharmacy, Mersin University, Mersin, TR 33169, Turkey;; 3 Department of Microbiology, Faculty of Medicine, Mustafa Kemal University, Hatay, TR 31040, Turkey; E-mail: nizamduran@hotmail.com (N. D.); 4 Health School, Mersin University, Mersin, TR 33169, Turkey; E-mail: gulay_borekci@yahoo.com (G. B.)

**Keywords:** Thiourea derivatives, Complexes, Antibacterial activity, Antifungal activity, *In vitro* studies, Lipophilicity

## Abstract

Five thiourea derivative ligands and their Ni^2+^ and Cu^2+^ complexes have been synthesized. The compounds were screened for their *in vitro* anti-bacterial activity using Gram-positive bacteria (two different standard strains of *Staphylococcus aureus*, *Staphylococcus epidermidis*, *Enterococcus faecalis*, *Streptococcus pyogenes*, *Bacillus cereus*) and Gram-negative bacteria (*Esherichia coli*, *Pseudomonas aeruginosa*, *Enterobacter cloacae*, *Proteus vulgaris*, *Enterobacter aerogenes*) and *in vitro* anti-yeast activity (*Candida albicans*, *Candida krusei*, *Candida glabrata*, *Candida tropicalis*, *Candida parapsilosis*). The minimum inhibitory concentration was determined for all ligands and their complexes. *In vitro* anti-yeast activity of both ligands and their metal complexes is greater than their *in vitro* anti-bacterial activity. The effect of the structure of the investigated compounds on the antimicrobial activity is discussed.

## Introduction

Industrial production and the use of Fe, Co, Cu, Ni, Zn, Cd, and Pb elements can cause environmental pollution. On the other hand, some of these metals are present in trace amounts as essential elements for biological systems and these metal ions also play an important role in bioinorganic chemistry. In order to understand the role of these metal ions in biological systems, structural studies of the biological compounds and their metal complexes are extremely important.

Compounds containing carbonyl and thiocarbonyl groups occupy an important position among organic reagents as potential donor ligands for transition metal ions [1,2,3,4,5,6,7]. Among these thiourea derivatives are potentially very versatile ligands, able to coordinate to a range of metal centres as neutral ligands, monoanions or dianions [1,2,3,4,5,6,7,8,9,10,11,12]. The oxygen, nitrogen and sulfur donor atoms of thiourea derivatives provide a multitude of bonding possibilities. Both the ligands and their metal complexes display a wide range of biological activity including antibacterial, antifungal, antitubercular, antithroid, antihelmintic, rodenticidal, insecticidal, herbicidal, and plant-growth regulator properties [13,14,15,16,17]. 

In view of this, our team focused on the synthesis, characterization, crystal structure, thermal behavior and antimicrobial activity of new thiourea derivatives [1,2,3,4,5,6,7,18,19,20,21,22,23,24,25]. Following our examination of the antimicrobial activity of thiourea derivative ligands and their metal complexes, we now report on the anti-bacterial and anti-yeast activity of five thiourea derivative ligands: (*N*-(diethylcarbamothioyl)cyclohexanecarboxamide [L^1^], *N*-(di-*n*-propylcarbamothioyl)cyclohexane carboxamide [L^2^] di-*n*-butylcarbamothioyl)cyclohexanecarboxamide [L^3^], *N*-(diphenylcarbamothioyl) cyclohexanecarboxamide [L^4^], *N*-(morpholine-4-carbonothioyl)cyclohexanecarboxamide [L^5^]) and their Ni^2+^ and Cu^2+^ metal complexes against standard bacterial (two different standard strains of *Staphylococcus aureus*, *Staphylococcus epidermidis*, *Enterococcus faecalis*, *Streptococcus pyogenes*, *Bacillus cereus*, *Esherichia coli*, *Pseudomonas aeruginosa*, *Enterobacter cloacae*, *Proteus vulgaris*, *Enterobacter aerogenes*) and yeast strains (*Candida albicans*, *Candida krusei*, *Candida glabrata*, *Candida tropicalis*, *Candida parapsilosis*).

## Results and Discussion

**Scheme 1:**
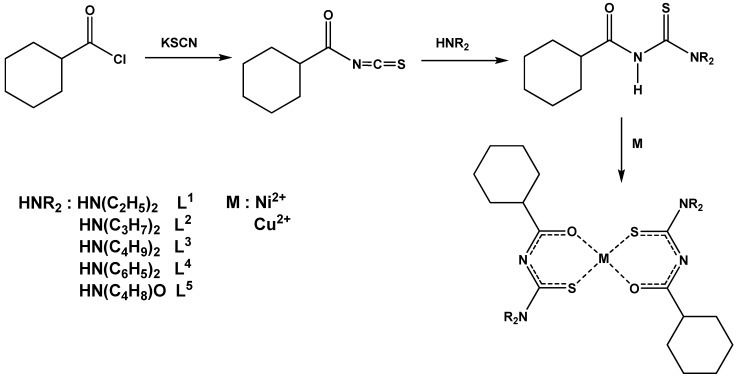
Synthesis of the compounds.

The syntheses (Scheme 1) involve the reaction of a cyclohexanecarbonyl chloride with potassium thiocyanate in acetone, followed by condensation of the resulting cyclohexanecarbonyl isothiocyanate with an appropriate secondary amine (diethylamine, di-*n*-propylamine, di-*n*-butylamine, diphenylamine ormorpholine). The ligands were purified by re-crystallization from an ethanol-dichloromethane mixture (1:2). The reaction of the ligands with metallic salts at room temperature with ethanol as solvent yielded the related metal complexes.

In the light of interesting antimicrobial activities of the coordination complexes, the thiourea derivative ligands and their Ni^2+^ and Cu^2+^ metal complexes were screened for antibacterial and antifungal activity against *S. aureus*, *S. epidermidis*, *E. faecalis*, *S. pyogenes*, *B. cereus*, *B. cereus*, *E. coli*, *P. aeruginosa*, *E. cloacae*, *P. vulgaris*, *E. aerogenes*, *C. albicans*, *C. krusei*, *C. glabrata*, *C. tropicalis*, *C. parapsilosis* by the broth microdilution procedure. The Gram positive anti-bacterial agent, amikacin, the Gram negative anti-bacterial agent, gentamycin, and the anti-fungal agent, nystatin, were used as controls. The *in vitro* antimicrobial properties against a number of Gram positive and Gram negative bacteria, and yeasts of both ligands and their metal complexes are presented in Table 1, Table 2 and Table 3, respectively.

**Table 1:** MIC values (μg/cm^3^) of the compounds against the tested Gram positive bacteria.

Compound	*S. aureus* (ATCC 25923)	*S. aureus* (ATCC 29213)	*S. epidermidis* (ATCC 12228)	*E. faecalis* (ATCC 29212)	*S. pyogenes* (Clinical isolate)	*B. cereus* (Clinical isolate)
**L^1^**	200	200	100	400	200	200
**L^2^**	400	200	200	200	200	100
**L^3^**	200	400	200	200	400	200
**L^4^**	100	100	100	100	200	100
**L^5^**	400	200	100	100	200	200
**NiL^1^_2_**	200	200	200	200	200	200
**NiL^2^_2_**	200	200	200	200	100	200
**NiL^3^_2_**	200	400	100	200	100	200
**NiL^4^_2_**	400	400	100	200	100	100
**NiL^5^_2_**	200	200	200	400	200	100
**CuL^1^_2_**	100	200	100	200	400	100
**CuL^2^_2_**	100	200	200	200	400	100
**CuL^3^_2_**	200	400	200	200	400	200
**CuL^4^_2_**	200	200	200	400	400	200
**CuL^5^_2_**	50	100	100	200	200	100
**Amikacin**	2	2	0.5	4	1	2

**Table 2:** MIC values (μg/cm^3^) of the compounds against the tested Gram negative bacteria.

Compound	*E. coli* (ATCC 25922)	*P. aeruginosa* (ATCC 27853)	*E. cloacae* (ATCC 13047)	*P. vulgaris* (ATCC 13315)	*E. aerogenes* (Clinical isolate)
**L^1^**	400	400	400	200	200
**L^2^**	200	400	200	200	400
**L^3^**	200	400	200	200	200
**L^4^**	200	400	200	200	400
**L^5^**	200	400	400	200	400
**NiL^1^_2_**	200	400	200	200	400
**NiL^2^_2_**	200	400	200	200	200
**NiL^3^_2_**	400	400	400	200	400
**NiL^4^_2_**	400	400	400	400	200
**NiL^5^_2_**	200	400	400	400	400
**CuL^1^_2_**	200	400	400	400	200
**CuL^2^_2_**	400	400	200	200	200
**CuL^3^_2_**	400	400	200	100	400
**CuL^4^_2_**	400	400	400	200	400
**CuL^5^_2_**	200	400	400	400	200
**Gentamycin**	0.5	1	2	2	0.5

**Table 3:** MIC values (μg/cm^3^) of the compounds against the tested fungi.

Compound	*C. albicans* (ATCC 90028)	*C. krusei* (ATCC 6258)	*C. glabrata* (ATCC 32554)	*C. tropicalis* (ATCC 20336)	*C. parapsilosis* (Clinical isolate)
**L^1^**	50	50	50	50	50
**L^2^**	50	50	25	25	100
**L^3^**	100	100	25	25	50
**L^4^**	25	50	50	50	50
**L^5^**	50	50	50	50	50
**NiL^1^_2_**	25	50	50	100	25
**NiL^2^_2_**	25	50	25	50	50
**NiL^3^_2_**	100	100	50	100	50
**NiL^4^_2_**	50	25	25	50	100
**NiL^5^_2_**	50	50	50	50	50
**CuL^1^_2_**	25	50	25	50	50
**CuL^2^_2_**	25	50	25	25	25
**CuL^3^_2_**	25	50	50	50	50
**CuL^4^_2_**	50	25	25	25	50
**CuL^5^_2_**	25	25	25	25	25
**Nystatin**	1	0.5	2	4	4

All compounds inhibited the growth of bacteria with MIC values ranging between 50 and 400 μg/cm^3^ and showed anti-yeast activity with MICs between 25 and 100 μg/cm^3^. According to the antimicrobial studies, all compounds showed such activity, albeit lower than their anti-yeast efficacy. This difference may be due to the differences between the cell structures of bacteria and yeast. While the cell walls of fungi contain chitin, the cell walls of bacteria contain murein [17]. In addition, fungi contain ergosterol in their cell membranes instead of the cholesterol found in the cell membranes of animals [4,26].

CuL^5^_2_ show good activity against *C. albicans*, *C. krusei*, *C. glabrata*, and *C. tropicalis*. NiL^3^_2_ showed low activity against *C. albicans*, *C. krusei*, and *C. tropicalis*. When all the anti-yeast MIC values are compared, twelve of fifteen compounds show good activity against *C. glabrata* and ten of fifteen compounds show low activity against *C. parapsilosis*. According to the anti-bacterial studies, the efficacy against Gram positive bacteria is higher than against Gram negative bacteria. Eleven of fifteen compounds show good activity against *S. epidermidis* and eight of fifteen compounds show low activity against *S. pyogenes*. In addition, L^4^ showed high activity against all Gram positive bacteria. 

The investigated compound antimicrobial activity values in this research were lower than that reported for other thiourea derivatives [4,6,17,19]. The main difference in the thiourea derivatives reported in this paper is the presence of the cyclohexyl moiety. The other derivatives included substituted benzyl groups. Lipophilicity, which correlates well with the bioactivity of chemicals, is a very important molecular descriptor and different lipophilic behaviour of compounds plays an important role in their biological activity mechanisms. The *n*-octanol/water partition coefficient (log *P*_ow_) is widely used as a general measure of lipophilicity. Compounds with benzyl groups have relatively higher log *P*_ow_ values and hence show more lipophilic character as compared to the compounds with cyclohexyl groups [27].

It is interesting to note that the investigated compounds with cyclohexane group show lower anti-microbial activity. This behavior can be attributed to the fact that due to their low lipophilicity, these compounds do not penetrate into the microorganisms as easily as the thiourea derivatives with benzyl group do. Similar behavior is observed in the anti-yeast activity MIC values for CuL^5^_2_ and NiL^5^_2_. The compound with a morpholine ring, which has the log *P*_ow_ value of 2.55, show higher anti-microbial activity than the other investigated compounds due probability to their higher lipophilic character.

The results show that the copper complexes are more active against the tested yeast as compared to the nickel complexes. The increase in antifungal activity of the copper complexes can be ascribed to the effect of the copper metal ion on the normal cell process. The complexation reaction reduces the polarity of the metal ion by the partial sharing of metal ion’s positive charge with donor groups and electron delocalization over the chelate ring. Thus, the lipophilic character of the central metal atom is enhanced which results in a higher capability to penetrate the microorganisms through the lipid layer of the cell membrane. Although MIC values for some compounds are good, unfortunately, the anti-yeast and anti-bacterial activity values of all tested compounds are lower than the reference compounds, thus these compounds cannot be suggested for clinical use. 

## Experimental

### Synthesis

The ligands were prepared by a procedure similar to that reported in the literature (Scheme 1) [3]. A solution of cyclohexanecarbonyl chloride (0.005 mole) in acetone (30 mL) was added dropwise to a suspension of potassium thiocyanate (0.005 mole) in acetone (30 mL). The reaction mixture was heated (50 °C) under reflux for 30 min, and then cooled to room temperature. A solution of secondary amine (0.005 mole) in acetone (30 mL) was added and the resulting mixture was stirred for 2 h. Hydrochloric acid (0.1 N, 100 mL) was added and the solution filtered. The solid product was washed with water and purified by recrystallization from an ethanol-dichloromethane mixture (1:2). Metallic complexes were prepared according to the method described in the literature [3]. A solution of the metallic acetate (0.01mole) in ethanol (30 mL) was added dropwise to a solution of the ligand in a 1:2 ratio for all metal with a small excess of ligand in ethanol (30 mL) at room temperature and the resulting mixture was stirred for 30 min. The solid complexes were filtered and re-crystallized from a 1:2 ethanol-dichloromethane mixture.

### Antimicrobial activity

The compounds were screened for their *in vitro* anti-bacterial and anti-yeast activities. Antimicrobial activities of both ligands and complexes were determined by the broth microdilution procedures and principles of the Clinical and Laboratory Standards Institute (CLSI) [28,29]. Minimal inhibitory concentrations for each compound were investigated against standard bacterial strains; *S. aureus* (ATCC 25923), *S. aureus* (ATCC 29213), *S. epidermidis* (ATCC 12228), *E. faecalis* (ATCC 29212), *S. pyogenes* (clinical isolate), *B. cereus* (clinical isolate), *E. coli* (ATCC 25922), *P. aeruginosa* (ATCC 27853), *E. cloacae* (ATCC 13047), *P. vulgaris* (ATCC 13315), *E. aerogenes* (clinical isolate), and yeast-like fungi; *C. albicans* (ATCC 90028), *C. krusei* (ATCC 6258), *C. glabrata* (ATCC 32554), *C. tropicalis* (ATCC 20336), *C. parapsilosis* (clinical isolate) obtained from the Refik Saydam Hıfzıssıhha Institute, Ankara, Turkey, Microbiology Culture Collection of Inonu University, Malatya, Turkey, and Department of Microbiology, Faculty of Medicine, Ege University, İzmir, Turkey. Bacterial and fungal colonies of the test organisms were suspended directly into a small volume of 0.9% saline and further diluted until turbidity matched the Mc Farland standard no: 0.5 Petri dishes containing Mueller-Hinton agar for bacteria and Sabouraud Dextrose agar for fungi were impregnated with these microbial suspensions. The stock solutions were prepared in dimethyl sulfoxide (DMSO), which had no effect on the microorganisms in the concentrations studied. All of the dilutions were done with distillated water. The concentrations of tested compounds were 400, 200, 100, 50, 25, 12.5, 6.25, 3.125 μg/cm^3^. DMSO was used as negative control. Amikacin, gentamycin, and nystatin were used as reference drugs for Gram positive anti-bacterial activity, Gram negative anti-bacterial activity and antifungal activity, respectively. All the inoculated plates were incubated at 35 °C and results were evaluated after 16-20 h for bacteria and 48 h for fungi. The lowest concentration of the compounds that prevented visible growth was considered to be minimal inhibitor concentrations (MICs).
